# Patients' perceived quality of the care provided during the management of HIV/AIDS in a tertiary care setting in Nigeria

**DOI:** 10.4314/ahs.v23i4.27

**Published:** 2023-12

**Authors:** Unyime Israel Eshiet, Chikosolu Njoku, Chidinma Wogu

**Affiliations:** Department of Clinical Pharmacy and Biopharmacy, University of Uyo, Nigeria

**Keywords:** Patient-centred care, HIV/AIDS, antiretroviral drugs, knowledge, attitude

## Abstract

**Background:**

The provision of patient-centred care by clinicians is believed to improve patient satisfaction with care as well as the outcome of treatment.

**Objective:**

To assess the quality of personalized care provided to people living with HIV/AIDS in a typical Nigerian health care setting and its association with patients' knowledge and attitude towards HIV/AIDS and patients' knowledge and attitude towards antiretroviral therapy.

**Methods:**

The study was a cross sectional study carried out in the HIV/AIDS clinic of the University of Uyo Teaching Hospital, Nigeria. Data on the demographic and clinical details of the patients were obtained from patients' case notes using a suitably designed, pre-piloted data collection instrument. Furthermore, data on the quality of patient-centred care, patients' knowledge and attitude towards HIV/AIDS, and patients' knowledge and attitude towards antiretroviral therapy was obtained using ‘Patient Assessment of Quality of Individualized care for Chronic Illness Scale’, ‘Patient knowledge and attitude towards HIV/AIDS questionnaire; and ‘Patient knowledge and attitude towards antiretroviral therapy questionnaire’, respectively.

Quantitative data were analysed using Statistical Program and Service Solutions (SPSS) version 25.0 computer package. Descriptive statistics were used to summarize data, while inferential statistics were used where applicable with statistical significance set at p<0.05.

**Results:**

The overall mean patients' satisfaction with individualized care score was 3.54 (SD = ±0.86; Max. = 5). The mean scores of the patients' knowledge and attitude towards HIV/AIDS were 6.80 (SD = ± 1.079; Max. = 8) and 5.5 (SD = ± 0.924; Max. = 7) respectively, while the mean scores of the patients' knowledge and attitude toward antiretroviral therapy were 5.7 (SD = ± 1.103; Max. = 10) and 4.2 (SD = ± 0.874; Max. = 6). Multivariate linear regression revealed that the quality of patient centred care was a predictor of knowledge and attitude towards antiretroviral therapy (B=0.511; CI = 95%, p = 0.024).

**Conclusion:**

The quality of patient-centred care provided to persons living with HIV/AIDS in the facility is satisfactory. Patients perceived quality of care appear to be a predictor of knowledge and attitude towards antiretroviral therapy.

## Introduction

A health system that provides patient-centred care is one that supports patients to make informed decisions about their own healthcare, and to successfully manage their condition. To achieve this, the provision of healthcare services must be in collaboration with the patients, and take into consideration patients' individual abilities, lifestyles, preferences and goals 1, 2.

Patient-centred care entails working together with the patient to develop and implement suitable therapeutic interventions to address individual health care needs. It is not just about giving people whatever they want or simply providing information 3. The healthcare provider is compassionate, respectful and considers the patient's views in making therapeutic decisions. In conventional healthcare delivery, patients are expected to abide by the practices and regimen that health care providers consider most suitable. In order to be considered patient-centred, healthcare delivery services need to be more accommodating and flexible to meet individual patient's needs in a manner that is convenient and suitable for them as practically possible. To this end, clinicians must work with patients and families to find the most appropriate way to provide their care 3, 4. The clinician-patient partnership can occur on a one-on-one basis, where each patient is involved in taking decisions about his/her healthcare; or on as a group of patients where the patients are collectively involved in taking decisions regarding the design and delivery of health care services. In both circumstances, the underlying philosophy is the same, that is, working with the patient to provide therapeutic care 3, 5.

Patient-centred care has been identified as a key component in the provision of high-quality healthcare. It is believed that putting patients in the centre of their care will improve the quality of the healthcare services provided, improve patient involvement in the care process, and reduce the burden on the health system 6 - 9.

HIV/AIDS is a major social and public health problem globally. It affects all age groups and is the leading cause of morbidity and mortality in sub-Saharan Africa 10. Among persons living with HIV, the knowledge regarding HIV and its transmission is essential to reduce the risk of superinfection and also prevent the transmission of the disease 11 - 14. Knowledge about HIV/AIDS has been identified as a powerful tool to prevent the transmission of the disease.

Effective HIV/AIDS care requires treatment with antiretroviral drugs. Adherence to antiretroviral drugs is influenced by a number of factors including knowledge regarding its use, and risks of non-adherence. Research findings have shown that PLHIV with sufficient knowledge of HIV and its management have improved adherence to antiretroviral therapy 15 - 16.

It is widely believed that the provision of patient-centred care by clinicians would improve patient satisfaction with care as well as the outcomes of treatment. This is because it focuses on patients' individual health needs by getting to know them as a person and recognizing their individuality 1, 4, 5. An assessment of patients' perception of the quality of personalized care they receive from their healthcare providers can serve as an audit for health care systems. This study was undertaken to assess the quality of personalized care provided to PLHIV in a typical Nigerian health care setting, and its association with patients' knowledge and attitude towards HIV/AIDS and patients' knowledge and attitude towards antiretroviral therapy.

## Methods

### Research setting

This study was carried at the University of Uyo Teaching Hospital (UUTH), in Uyo, Nigeria.

UUTH is a tertiary health care facility with medical residents located in Uyo, Nigeria. UUTH also provides primary and secondary health care services. It is an affiliate of the University of Uyo and is a major referral centre for tertiary health care service within the region.

### Research design

This study was a cross sectional study using suitably designed and validated instruments to extract data from patients living with HIV/AIDS receiving treatment at University of Uyo Teaching Hospital (UUTH), in Uyo, Nigeria. Patients living with HIV/AIDS were recruited from the antiretroviral (ARV) clinic of the hospital.

### Research/data collection instruments

Data on the demographic and clinical characteristics of the patients were obtained from patients' case notes using a suitably designed, pre-piloted data collection instrument. Data that was collected from the patients and their case notes included: patient's gender, patient's age, educational level, duration of illness, presence of co-morbidity, type of co-morbidity (if present).

Furthermore, data on the quality of patient-centred care, patients' knowledge and attitude towards HIV/AIDS, and patients' knowledge and attitude towards antiretroviral therapy was obtained using ‘Patient Assessment of Quality of Individualized care for Chronic Illness Scale’, ‘Patient knowledge and attitude towards HIV/AIDS questionnaire, and ‘Patient knowledge and attitude towards antiretroviral therapy questionnaire’, respectively.

### Patient assessment of quality of individualized care for chronic illness scale

The Patient Assessment of Quality of Individualized Care for Chronic Illness Scale is a 5-Item self-administered questionnaire with a ‘yes or no’ response option, designed by the researchers from literature searches 1, 17, 20, validated and used to evaluate the quality of patient-centred care provided.

The developed questionnaire was reviewed by a team of expert panel composed of clinicians practicing in the academia, hospital and community. This was done to confirm content validity. These experts reviewed the questionnaires individually and rated them based on four categories: content relevance, clarity, simplicity and ambiguity. All the comments from the content and face validation were thoroughly discussed by the research team. The items were either edited, removed or remained unchanged after extensive discussion among the researchers. Furthermore, a pilot test was carried out on the revised questionnaire to assess the readability and general formatting of the questionnaire. This was done using 20 randomly selected patients living with HIV/AIDS.

The higher the score on the patient assessment scale, the higher the quality of individualized care provided (a ‘yes’ response to each question on the scale attracted a score). Furthermore, a scoring template was used to grade the patient assessment scores into different levels of individualized care with scores of <3 indicating a poor level of individualized care, and scores of 3 and ≥ 4 indicating a moderate and high level of individualized care respectively.

### Patient knowledge and attitude toward HIV/AIDS questionnaire

This is a 15-item instrument (8 questions assessing patients' knowledge of HIV/AIDS and 7 questions assessing patients' attitude towards HIV/AIDS) adopted from a previous study conducted among PLHIV receiving care at the University college hospital, Ibadan [20]. It was used in this study to assess the knowledge and attitude towards HIV/AIDS among the study participants.

Patients' knowledge of HIV/AIDS was graded based on their responses to the questions that assessed their knowledge of HIV/AIDS (each correct response attracted a score). Patients with scores < 4 were graded as having a poor knowledge of HIV/AIDS, while those who scored 4 - 5, and those who scored above 5 were graded as having a moderate and high knowledge of HIV/AIDS respectively.

### Patient knowledge and attitude towards antiretroviral therapy questionnaire

This is a 16-item instrument (10 questions assessing patients' knowledge of antiretroviral therapy and 6 questions assessing patients' attitude towards antiretroviral therapy) adapted from a previous study conducted among PLHIV receiving care at the University college hospital, Ibadan 10. It was used in this study to assess the knowledge and attitude towards antiretroviral therapy among the study participants.

Patients' knowledge of antiretroviral therapy was graded based on their responses to the questions that assessed their knowledge of antiretroviral therapy (each correct response attracted a score). Patients with scores < 5 were graded as having a poor knowledge and attitude antiretroviral therapy, while those who scored 5 - 6, and those who scored above 6 were graded as having a moderate and high knowledge of antiretroviral therapy respectively.

### Study population/sample size

#### Eligibility criteria

All patients with HIV/AIDS who met the eligibility criteria were recruited into the study.

The eligibility criteria for recruitment into the study were;

- Patients diagnosed with HIV/AIDS and receiving treatment at UUTH within the period of the study;
- Patients who expressed willingness to participate in the study;
- Patients who provided a written informed consent to participate in the study.

#### Exclusion criteria

The exclusion criteria were;

- Patients less than 16 years old.
- Patients with active psychiatric illnesses.
- Patients who were not able to communicate effectively in English language.

Sample size was determined by using the formula described by Yamane 21.


$$n=N/1+N\;(e^{2})$$

Where n = calculated sample size; N = Population of HIV/AIDS patients that attended clinic within the period of the study; e = level of precision (+ 5%).

The average daily clinic attendance at the antiretroviral clinic (ARV) of the UUTH is placed at 75 patients. The duration of data collection was 8 weeks. Based on this estimate, the projected population of HIV/AIDS patients that was expected to attend clinic within the period of study was about 3,000 patients. Hence, the calculated sample size for this study using the formula described above was 353 patients. However, four hundred patients, who met the eligibility criteria were successfully interviewed.

### Data analysis

Data were analysed using the IBM Statistical Products and Services Solutions (SPSS) for Windows, version 25.0 (IBM Corp, version 25.0 Armonk, NY and USA). Frequencies and means were used to summarize descriptive statistics. Correlation and multivariate linear regression analysis were used to test the relationship between both assessment variables. Statistical significance was set at p < 0.05.

## Results

Demographic and clinical characteristics of the patients Four hundred patients with HIV/AIDS who met the inclusion criteria were recruited into the study. The demographic and clinical characteristics of the patients are as presented in Table 1.

**Table 1:** Demographic/clinical characteristics of the patients

Characteristics	Frequency	Percent (%)
**Gender**		
Male	127	31.75
Female	273	68.25
**Age (years)**		
19 – 25	16	4.00
26 – 35	86	21.50
36 – 45	144	36.00
46 - 55	93	23.25
56 – 65	48	12.00
>65	13	3.35
**Educational Level**		
Primary	72	18.00
Secondary	185	46.25
Tertiary	143	35.75
**Religion**		
Christianity	393	98.25
Islam	7	1.75
**Marital Status**		
Single	122	30.50
Married	213	53.25
Separated	16	4.00
Widowed	49	12.25
**Duration of Illness**		
<1 year	24	6.00
1 – 5 years	120	30.00
6 – 10 years	111	27.75
11 – 15 years	113	28.25
16 – 20 years	29	7.25
>20 years	3	0.75
**Presence of Co-morbidity**		
None	305	76.25
Yes	95	23.75
**Type of Co-morbidity**		
Hypertension (HTN)	70	73.68
Diabetes Mellitus	9	9.47
HTN & DM	6	6.32
Others	10	10.53

About 36% of the patients had been receiving care for HIV/AIDS for over 10 years, while about 24% of the patients had other comorbidities with hypertension being the most frequently reported comorbidity in this population.

### Patients' assessment of quality of individualized care

About 90% of the patients with HIV/AIDS were satisfied with the individualized care they received from their healthcare providers, and 90% of the patients affirmed that their healthcare providers knew and treated them as a person not just as a patient.

The item-by-item mean patients' assessment scores for patient centred care based on the Patient Assessment of Quality of Individualized Care for Chronic Illness scale is as presented in Table 2.

**Table 2:** Patient assessment of quality of individualized care

S/N	Questions on the Scale	Yes	No
1.	My healthcare providers in this clinic know me as a person.	361 (90%)	39 (10%)
2.	My healthcare providers in this clinic are genuinely interested in my health and general well-being.	380 (95%)	20 (5%)
3.	My healthcare providers in this clinic usually ask for my ideas/suggestions before initiating a treatment plan.	239 (60%)	161 (40%)
4.	My healthcare providers in this clinic encourage me to talk	356 (89%)	44 (11%)
5.	about any problems with my medicines or their effects. I am satisfied with the care provided by my healthcare providers in this clinic.	360 (90%)	40 (10%)

The overall mean patient assessment of individualized care score was 3.54 (±0.86).

Based on the deduced patients' assessment of patient centred care level, where patient assessment scores of < 3 was considered a low level of personalized care, patient assessment scores < 3 and ≥ 4 were considered moderate and high levels of personalized care respectively, we found that in 8.5 % (34) of the cases studied, a low level of personalized care was provided, 23% (92) of the cases had a moderate level of personalized care, while 68.5% (274) of the patients had a high level of personalized care.

### Patients' knowledge and attitude toward HIV/AIDS

The mean scores of the patients' knowledge and attitude towards HIV/AIDS were 6.80 (SD = ± 1.079; Max. = 8) and 5.5 (SD = ± 0.924; Max. = 7) respectively. The item-by-item responses of the patients to the knowledge and attitude toward HIV/AIDS questionnaire are presented in Table 3 below.

**Table 3:** Patients' knowledge and attitude towards HIV/AIDS questionnaire

Knowledge of HIV/AIDS (*Expected answer*)	Yes	No
HIV/AIDS is a disease without a cure (*Yes*)	245 (61.3%)	155 (38.7%)
HIV/AIDS is an invention to scare people (*No*)	81 (20.2%)	319 (79.8%)
A healthy-looking person cannot have HIV/AIDS (*No*)	61 (15.2%)	339 (84.8%)
Proper condom use can be protective against HIV/AIDS (*Yes*)	360 (90%)	40 (10%)
HIV transmission can occur from mother to child (*Yes*)	346 (86.5%)	54 (13.5%)
An unscreened blood transfusion can result in HIV/AIDS (*Yes*)	364 (91%)	36 (9%)
Unprotected sexual intercourse between a man and woman can result in HIV/AIDS (*Yes*)	379 (94.8%)	21 (5.2%)
Multiple sexual partners increase the risk of HIV infection (*Yes*)	368 (92%)	32 (8%)
**Attitude toward HIV/AIDS (*Expected answer*)**		
A person with HIV/AIDS has no hope (*No*)	44 (11%)	356 (89%)
A person with HIV should have sexual intercourse without a condom (*No*)	52 (13%)	348 (87%)
Having HIV is not the end of one's life (*Yes*)	359 (89.8%)	41 (10.2%)
A person with HIV can get married and have children (*Yes*)	375 (93.8%)	25 (6.2%)
A person with HIV should not aspire to be an achiever (*No*)	63 (15.7%)	337 (84.3%)
HIV/AIDS is a punishment for immoral behaviour (*No*)	76 (19%)	324 (81%)
It is shameful to have HIV/AIDS (*No*)	132 (33%)	268 (67%)

Fourteen (3.5%) of the patients had a poor knowledge of HIV/AIDS, while 53 (13.25%) and 333 (83.25%) of the patients had a moderate and high knowledge of HIV/ AIDS respectively.

### Patients' knowledge and attitude toward antiretroviral therapy

The mean scores of the patients' knowledge and attitude toward antiretroviral therapy were 5.7 (SD = ± 1.103; Max. = 10) and 4.2 (SD = ± 0.874; Max. = 6). The item-by-item responses of the patients to the knowledge and attitude toward ART questionnaire are presented in Table 4 below.

**Table 4:** Patients' knowledge and attitude towards antiretroviral therapy questionnaire

Knowledge of ART (*Expected answer*)	Yes	No
ART consists of drugs to cure HIV/AIDS (*No*)	101 (25.2%)	299 (74.8%)
ART consists of drugs to suppress the activity of HIV (*Yes*)	375 (93.8%)	25 (6.2%)
CD4 count is the number of HIV viruses in the blood (*No*)	47 (11.8%)	353 (88.2%)
CD4 count is the number of body soldiers (*Yes*)	270 (67.5%)	130 (32.5%)
Viral load is the number of HIV viruses in the blood (*Yes*)	148 (37%)	252 (63%)
Viral load is the number of body soldiers (*No*)	344 (86%)	56 (14%)
ART increases the viral load (*No*)	56 (14%)	344 (86%)
ART increases the CD4 count (*Yes*)	99 (24.8%)	301 (75.3%)
ART reduces the viral load (*Yes*)	361 (90.3%)	39 (9.7%)
ART reduces the CD4 count (*No*)	322 (80.5%)	78 (19.5%)
		
**Attitude towards ART (*Expected answer*)**		
I do not need ARV drugs because I am not convinced that I have HIV/AIDS (*No*)	32 (8%)	368 (92%)
Because there is no cure for HIV, taking the drugs is a waste of time (*No*)	19 (4.7%)	381 (95.3%)
Taking ARV drugs for one's life time is tiring (*No*)	115 (28.7%)	285 (71.3%)
ARV drugs help to prolong lives (*Yes*)	373 (93.3%)	27 (6.8%)
You should take HIV drugs only when you feel sick (*No*)	22 (5.5%)	378 (94.5%)
It is shameful to be on ARV therapy (*No*)	67 (16.7%)	333 (83.3%)

Sixteen (4.0%) of the patients had a poor knowledge and attitude toward ART, while 249 (62.25%) and 135 (33.75%) of the patients had a moderate and high knowledge and attitude toward ART.

### Test of rela tionship between the quality of patient centred care and humanistic outcome assessment variables

Correlation analysis was conducted to evaluate the relationship between the quality of patient centred care and humanistic outcome assessment variables. In this bivariate analysis, results showed that the quality of patient centred care was significantly positively correlated with patients' knowledge and attitude towards antiretroviral therapy (r = 0.168; p = 0.017) suggesting that as the quality of patient centred care improves, patient knowledge and attitude towards HAART would improve.

Although the bivariate analysis also found a positive correlation between the quality of patient centred care and patients' knowledge and attitude towards HIV/AIDS (r = 0.058; p = 0.414) the association was not significant.

Multivariate linear regression revealed that the quality of patient centred care was a predictor of knowledge and attitude towards antiretroviral therapy (B=0.511; CI = 95%; p = 0.024), i.e., every unit increase in the quality of patient centred care score improved patient knowledge and attitude towards antiretroviral therapy score by 0.511 units).

## Discussion

Patient-centred care is central to the goals and objectives of healthcare delivery. A Patient's opinion can be used as a key factor in selecting the mode of treatment and in delivering healthcare services. The quality of patient centred care provided in our study site for PLHIV as reported by the patients in this study was high. Patients' perception of care and inputs are critical in the overall improvement of quality healthcare delivery system 21-22.

Although majority of the study participants had moderate knowledge and attitude regarding HIV/AIDS, we found that a significant proportion of the patients had poor knowledge and attitude towards the condition. For instance, about one-fifth of the patients believed that HIV is an invention to scare people. Poor knowledge of HIV/AIDS among PLHIV is believed to be responsible for the high-risk behaviour among these population of patients and is a major setback for the ongoing interventions targeted at preventing the spread of the disease 11. Sufficient knowledge about HIV/AIDS, particularly amongst PLHIV is necessary to prevent transmission and reduce the risk of superinfection.

Stigmatization against PLHIV still poses a huge problem in the management of the condition. We observed that about a third of the patients studied considered living with HIV/AIDS shameful. Stigma and discrimination have been identified as major challenges affecting PLHIV globally due to their HIV status 23. They are major barriers to voluntary testing, treatment uptake, and adherence to medication. Although a 2017 review of policies and programs targeted at eliminating HIV stigmatization in Nigeria revealed that tremendous efforts have made towards this drive, the reviewers identified the need for more concerted contributions towards eliminating HIV stigmatization in the country. They recommended a strengthened design, planning, implementation, monitoring, and evaluation of context-specific stigma reduction programmes 24.

Although majority of the patient had an appreciable knowledge of antiretroviral therapy, we are concerned with some of the responses by a significant proportion of the patients regarding antiretroviral therapy. For instance, almost one-tenth of the patients were still not convinced that they had HIV, therefore believing they did not really need the antiretroviral drugs and about one-sixth of the patients considered taking antiretroviral drugs shameful. We find these assertions quite disturbing because such negative attitude towards HIV treatment would affect adherence, increasing the risk of drug resistance and thereby increasing the global burden of the disease.

A positive attitude and disposition towards HIV and antiretroviral therapy among patients is needed to achieve treatment goals. Effective patient education and counselling is required to achieve this. Patient beliefs about antiretroviral therapy has been found to be significantly associated with adherence 25. Reports indicate that improving the knowledge of the condition among PLHIV results in an improved adherence to antiretroviral therapy 15, 16. Clinicians must be aware of the need to continuously remind patients that HIV is a chronic condition that requires lifelong treatment. Most importantly, clinicians should endeavour to improve the perception of these patients regarding their condition by providing care in a more patient friendly and patient centred manner.

Although we found a positive correlation between the quality of patient-centred care (as judged by the patients) and patients' knowledge and attitude towards HIV/AIDS, this relationship was not statistically significant. On the other hand, a significant positive correlation between the quality of patient-centred care, as judged by the patient, and patients' knowledge and attitude towards antiretroviral therapy was found. Furthermore, multivariate analysis revealed that the quality of patient-centred care was a predictor of patients' knowledge and attitude towards antiretroviral drugs. Our findings are in consonance with the belief that the provision of patient-centred care improves outcomes in patients with chronic conditions. A review of studies examining the relationship between measures of patient-centred consulting and outcomes in primary care found links between person-centred clinician behaviour and selected patient health outcomes [26]. Personalized care is becoming increasingly considered a major component in the development and implementation of high-quality clinical care. It is a key indicator of quality of care and should be prioritized when implementing reforms targeted at improving healthcare delivery systems 27 - 30.

## Conclusion

The perceived quality of patient-centered care provided to persons living with HIV/AIDS in the facility seems satisfactory, and this appeared to be a predictor of knowledge and attitude towards antiretroviral therapy. There is a need to prioritize patients' involvement in the provision of care to persons living with HIV/AIDS.

## Study limitation

This study was cross-sectional and conducted in one tertiary HIV/AIDS treatment facility which may limit the generalizability of the research findings. Moreover, the use of non-probability sampling method in the recruitment of study participants and the possibility of social desirability bias among the participants may have affected the findings of the study.

## References

[1] Health Innovation Network South London What is patient-centred care and why is it important?.

[2] Sepucha K, Uzogarra B, O'Connor M (2008). Developing instruments to measure the quality of decisions: early results for a set of symptom-driven decisions. Patient Educ Counsel.

[3] The Health Foundation Patient centred care made simple.

[4] Gill PS (2013). Patient Engagement: An investigation at a primary care clinic. Int J Gen Med.

[5] Redrup Publications Introducing Person-Centred Care Approaches.

[6] Ashby ME, Dowding C (2001). Hospice care and patients' pain: communication between patients, relatives, nurses and doctors. Int J Pall Care Nurs.

[7] Dowsett SM, Saul JL, Butow PN, Dunn SM, Boyer MJ, Findlow R, Dunsmore J (2000). Communication styles in the cancer consultation: preferences for a patient-centred approach. Psycho-oncology.

[8] Kwan J, Sandercock P (2004). In hospital care pathways for stroke. Coch Database Sys Rev.

[9] Simces Z (2003). Exploring the link between public involvement/citizen engagement and quality health care. A review and analysis of the current literature.

[10] Olowookere SA, Fatiregun AA, Adewole IF (2012). Knowledge and attitudes regarding HIV/AIDS and antiretroviral therapy among patients at a Nigerian treatment clinic. J Infect Dev Ctries.

[11] Yathiraj AB, Unnikrishnan B, Ramapuram JT, Thapar R, Mithra P, Madi D, Kumar N, Kulkarni V, Holla R, Ambalavanan J, Darshan BB (2017). HIV-Related Knowledge among PLWHA Attending a Tertiary Care Hospital at Coastal South India - A Facility-Based Study. Journal of the International Association of Providers of AIDS Care.

[12] Avants SK, Warburton LA, Hawkins KA, Margolin A (2000). Continuation of high-risk behavior by HIV-positive drug users. Treatment implications. J Subst Abuse Treat.

[13] O'Mahony P, Barry M (1992). HIV risk of transmission behaviour amongst HIV-infected prisoners and its correlates. Br J Addict.

[14] Wingood GM, DiClemente RJ, Mikhail I, Lang DL, Mcrre DH, Davies SI, Hardin JW, Hook EW, Saag M (2004). A randomized controlled trial to reduce HIV transmission risk behaviors and sexually transmitted diseases among women living with HIV: The WiLLOW Program. J Acquir Immune DeficSyndr.

[15] Pradier C, Bentz L, Spire B, Tourette-Turgis C, Morin M, Souville M, Rebillon M, Fuzibet J, Pesce A, Dellamonica P, Moatti J (2003). Efficacy of an educational and counseling intervention on adherence to highly active antiretroviral therapy: French prospective controlled study. HIV Clin Trials.

[16] Goujard C, Bernard N, Sohier N, Peyramond D, Lancon F, Chwalow J, Arnould B, Delfraissy J (2003). Impact of a patient education program on adherence to HIV medication: a randomized clinical trial. J Acquir Immune Defic Syndr.

[17] Beach CM, Keruly J, Moore RD (2006). Is the Quality of the Patient-Provider Relationship Associated with Better Adherence and Health Outcomes for Patients with HIV?. J GEN INTERN MED.

[18] Schneider J, Kaplan SH, Greenfield S, Li W, Wilson IR (2004). Better Physician-Patient Relationships Are Associated with Higher Reported Adherence to Antiretroviral Therapy in Patients with HIV Infection. J GEN INTERN MED.

[19] Health Foundation Helping measure person-centred care - A review of evidence about commonly used approaches and tools used to help measure person-centred care.

[20] Wolf DM, Lehman L, Quinlin R, Zullo T, Hoffman L (2008). Effect of Patient-Centred Care on Patient Satisfaction and Quality of Care. J Nurs Care Qual.

[21] Greenfield G (2014). GPs should be rewarded for patient experience to encourage a person centred NHS. BMJ.

[22] Parish E (2015). BMJ roundtable debate: How can we get better at providing patient centred care?. BMJ.

[23] Fauk NK, Ward RP, Hawke K, Mwanri L (2021). HIV Stigma and Discrimination: Perspectives and Personal Experiences of Healthcare Providers in Yogyakarta and Belu, Indonesia. Front. Med.

[24] Odimegwu CO, Akinyemi JO, Alabi OO HIV-Stigma in Nigeria: Review of Research Studies, Policies, and Programmes. AIDS Research and Treatment.

[25] Schneider J, Kaplan SH, Greenfield S, Li W, Wilson IR (2004). Better Physician-Patient Relationships Are Associated with Higher Reported Adherence to Antiretroviral Therapy in Patients with HIV Infection. J GEN INTERN MED.

[26] van Zanten M, Boulet JR, Norcini JJ, McKinley D (2005). Using a standardised patient assessment to measure professional attributes. Med Educ.

[27] Ashby ME, Dowding C (2001). Hospice care and patients' pain: communication between patients, relatives, nurses and doctors. Int J Pall Care Nurs.

[28] Dowsett SM, Saul JL, Butow PN, Dunn SM, Boyer MJ, Findlow R, Dunsmore J (2000). Communication styles in the cancer consultation: preferences for a patient-centred approach. Psycho-oncology.

[29] Kwan J, Sandercock P (2004). In hospital care pathways for stroke. Coch Database Sys Rev.

[30] US Institute of Medicine (2001). Crossing The Quality Chasm: A New Health System for the 21st Century.

